# High-Dose versus Low-Dose Vitamin D Supplementation and Arterial Stiffness among Individuals with Prehypertension and Vitamin D Deficiency

**DOI:** 10.1155/2015/918968

**Published:** 2015-09-16

**Authors:** Amanda Zaleski, Gregory Panza, Heather Swales, Pankaj Arora, Christopher Newton-Cheh, Thomas Wang, Paul D. Thompson, Beth Taylor

**Affiliations:** ^1^Division of Cardiology, Hartford Hospital, Hartford, CT 06102, USA; ^2^Department of Kinesiology, University of Connecticut, Storrs, CT 06069, USA; ^3^Cardiology Division, Department of Medicine, University of Alabama, Birmingham, AL 16686, USA; ^4^Cardiovascular Research Center and Center for Human Genetic Research, Massachusetts General Hospital, Harvard Medical School, Boston, MA 02115, USA; ^5^Division of Cardiovascular Medicine, Vanderbilt University Medical Center, Nashville, TN 37201, USA

## Abstract

*Introduction*. Vitamin D deficiency is associated with the onset and progression of hypertension and cardiovascular disease (CVD). However, mechanisms underlying vitamin D deficiency-mediated increased risk of CVD remain unknown. We sought to examine the differential effect of high-dose versus low-dose vitamin D supplementation on markers of arterial stiffness among ~40 vitamin D deficient adults with prehypertension. *Methods*. Participants were randomized to high-dose (4000 IU/d) versus low-dose (400 IU/d) oral vitamin D3 for 6 months. 24 hr ambulatory blood pressure (BP), carotid-femoral pulse wave velocity, and pulse wave analyses were obtained at baseline and after 6 months of vitamin D supplementation. *Results*. There were no changes in resting BP or pulse wave velocity over 6 mo regardless of vitamin D dose (all *p* > 0.202). High-dose vitamin D decreased augmentation index and pressure by 12.3 ± 5.3% (*p* = 0.047) and 4.0 ± 1.5 mmHg (*p* = 0.02), respectively. However, these decreases in arterial stiffness were not associated with increases in serum 25-hydroxyvitamin D over 6 mo (*p* = 0.425). *Conclusion*. High-dose vitamin D supplementation appears to lower surrogate measures of arterial stiffness but not indices of central pulse wave velocity. *Clinical Trial Registration*. This trial is registered with www.clinicaltrials.gov (Unique Identifier: NCT01240512).

## 1. Introduction

Vitamin D deficiency is a major public health problem, affecting 33–58% of the US population [1, 2]. Low vitamin D status is associated with a myriad of negative health outcomes including poor musculoskeletal health [3, 4], cognitive decline [5], and cancer progression and mortality [6]. Of most recent interest is the association between vitamin D deficiency and major cardiovascular disease (CVD) risk factors, including hypertension [4, 7–9]. Indeed a meta-analysis of observational studies has shown that decreases in vitamin D by 16 ng/dL confer a 16% greater risk for hypertension [10]. Mechanisms underlying vitamin D deficiency-mediated increased risk of hypertension are not clear but may be related to* arterial stiffness*, a well-documented independent predictor of incident hypertension [11], CVD related events [12], and all-cause mortality [13, 14]. Interestingly, vitamin D receptors are expressed throughout the cardiovascular system, including endothelium and vascular smooth muscle cells [15, 16]. Therefore, it is possible that underlying changes in arterial stiffness may partially explain the reported associations among vitamin D status, blood pressure (BP), and CVD risk.

To date, though, clinical trials investigating the relationship between Vitamin D supplementation, BP, and/or arterial stiffness have been equivocal. For example, some trials have reported systolic BP (SBP) reductions ranging from ~5 to 13 mmHg [17–19], while others have shown no significant effect [20, 21]. These inconsistencies could be attributable to heterogeneity in vitamin D dosage, initial BP status, BP assessment, and study duration and quality. Similarly, the few trials looking at vitamin D, BP, and arterial stiffness have also been inconclusive, showing either a beneficial effect or lack of effect. Since such studies to date have been conducted in individuals on antihypertensive therapy and without vitamin D deficiency, the clinical interpretation of these results is questionable as the beneficial effects of vitamin D on cardiovascular outcomes may be observed independently or in combination with alterations to BP [19].

Therefore, the purpose of the current analysis was to examine the influence of high-dose and/or low-dose vitamin D supplementation on BP as well as indices of arterial stiffness. We hypothesized that normalization of vitamin D as a result of supplementation would yield significant reductions in arterial stiffness in a dose response manner.

## 2. Methods

The present study is part of a larger clinical trial, “*Vitamin D Therapy in Individuals with Prehypertension or Hypertension: The DAYLIGHT Trial*,” of which the methods have previously been published in detail (NCT01240512) [21]. DAYLIGHT is the largest prospective, double-blind, randomized, and controlled trial study designed to examine the influence of vitamin D on BP [21]. Briefly, 534 individuals (36 ± 10 yr) with untreated, elevated BP (131 ± 10 mmHg) and vitamin D deficiency (15 ± 6.3 ng/mL) were randomized to 6 months of low-dose (400 IU/d) or high-dose (4000 IU/d) vitamin D. The primary endpoint of DAYLIGHT was 24 hr BP, the gold standard of BP assessment [22]. Body Mass Index (BMI), season, sunlight exposure, alcohol intake, and smoking status were also collected as they may influence the effect of vitamin D on vascular function and BP changes from baseline. Participants were recruited at four sites: Massachusetts General Hospital, Boston, MA; Cultural Wellness Center, Minneapolis, MN; Abbott Northwestern Hospital, Minneapolis, MN; and Hartford Hospital, Hartford, CT. Of the four sites, Hartford Hospital was the only site to assess arterial stiffness before and after 6 mo of vitamin D supplementation as a secondary endpoint. The resultant substudy was performed in 41 individuals with similar characteristics to the main clinical trial (Table 1).

### 2.1. Inclusion and Exclusion Criteria

The main study enrolled individuals (18 to 50 yr) with clinic SBP >120 mmHg and 25-hydroxyvitamin D level ≤25 ng/mL at the screening visit. Individuals were excluded if SBP was greater than 160 mmHg and/or if DBP exceeded 99 mmHg. Individuals were also excluded if they had used any antihypertensive medication or vitamin D supplementation (>400 IU/d) in the past 3 mo or had any known CVD.

### 2.2. Vitamin D Supplementation

Participants were randomly assigned to once-daily doses of either 400 IU/d or 4000 IU/d oral vitamin D (cholecalciferol; Ddrops Co., Woodbridge, ON, Canada).

### 2.3. Blood Pressure Assessment

Clinic BP was assessed at the screening visit and at each office visit using a validated digital BP monitor (HEM-907X, Omron Healthcare, Inc., Bannockburn, IL) and according to standards set by the American Heart Association (AHA) [23]. 24 hr ambulatory BP was assessed at baseline and after 6 months (Spacelabs Healthcare, Issaquah, WA) with an appropriately sized cuff. Changes in clinic BP, mean 24 hr BP, and daytime and nighttime ambulatory BP were collected to explore the relation of vitamin D status to change in clinic and 24 hr BP.

### 2.4. Arterial Stiffness Assessment

Measurement of arterial stiffness parameters occurred following a 10 min supine rest period using the SphygmoCor CPV Central Blood Pressure/Pulse Wave Velocity System (Sydney, Australia). Pulse waveforms of the left carotid and left femoral artery were recorded sequentially by applanation tonometry to determine central pulse wave velocity (PWV). Pulse waveforms obtained over a 10 sec period at the left radial artery were used to determine indices of pulse wave analyses (PWA), subendocardial viability ratio (SEVR), augmentation pressure (AP), and augmentation index (AIx). Measures of PWV and PWA were performed at baseline and after 6 months of vitamin D supplementation.

### 2.5. Statistical Analysis

Differences in baseline characteristics between low-dose and high-dose vitamin D groups were assessed with a one-way analysis of variance (ANOVA). Two-way repeated measures ANOVA was used to determine differences due to vitamin D supplementation, time, and their interaction for arterial stiffness measures and BP. Linear regression was performed to examine the contribution of baseline values predicted changes in arterial stiffness, controlling for age and sex. Data are reported as mean ± standard error of the mean (SEM). All statistical analyses were performed using the Statistical Package for the Social Sciences (SPSS) 19.0 program for Windows (SPSS Inc., Chicago, IL) with *p* ≤ 0.05 considered as statistically significant.

## 3. Results

Baseline subject characteristics for the total sample (*n* = 41) are described in Table 1. Baseline parameters did not differ between groups with the exception that subjects in the high-dose group were slightly older than subjects in the low-dose group (Table 1; *p* < 0.05). Changes in safety laboratory measures (i.e., plasma calcium, creatinine, phosphorus, and transaminase) did not differ between the high-dose and low-dose vitamin D arms at 6 months.

### 3.1. Vitamin D Supplementation

**Figure 1:**
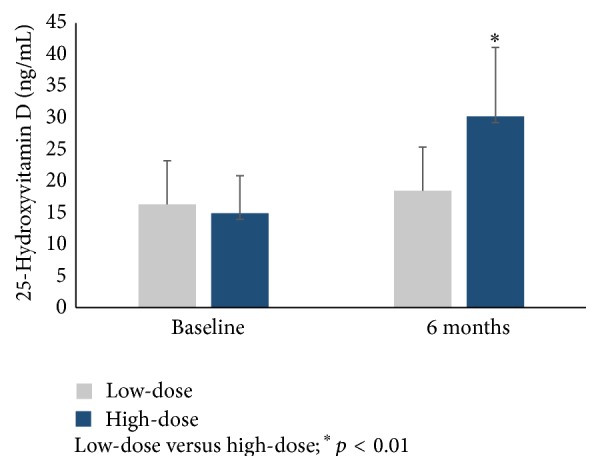
Serum 25-hydroxyvitamin D levels (±SD) before and after 6 months of high-dose versus low-dose supplementation.

Mean 25-hydroxyvitamin D levels at baseline did not differ between groups (mean, 15.7 ± 6.3 ng/mL; *p* ≥ 0.05), with 93% of the study sample <20 ng/mL, thus meeting the criteria for vitamin D deficiency [24]. Over the course of 6 months, subjects receiving low-dose (400 IU) versus high-dose vitamin D supplementation (4,000 IU) increased 25-hydroxyvitamin D by 4.4 ± 7.2 ng/mL and 16.0 ± 10.7 ng/mL, respectively (Figure 1; *p* < 0.01). At the end of the study, the proportions of individuals with 25-hydroxyvitamin D <20 ng/mL were 63% and 25% in the low-dose and high-dose groups, respectively.

### 3.2. Vitamin D Supplementation and Blood Pressure

**Figure 2:**
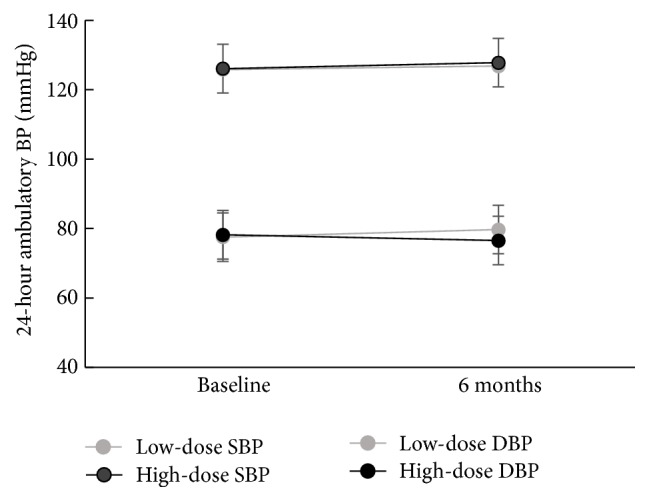
24 hr mean ambulatory blood pressure before and after 6 months of high-dose versus low-dose supplementation.

Baseline 25-hydroxyvitamin D was negatively correlated with baseline mean 24 hr SBP (*p* < 0.01) and 24 hr DBP (*p* < 0.05). Mean 24 hr SBP at baseline did not differ between groups (Figure 2). Similarly, mean 24 hr DBP at baseline did not differ between groups (Figure 2). Over the course of 6 months, there was no change in mean 24 hr BP after low-dose or high-dose vitamin D supplementation (Figure 2; *p* ≥ 0.05), consistent with the published findings from the main study [21]. Similarly, over the course of 6 months, there were no changes in clinic BP, daytime ambulatory BP, or nighttime ambulatory BP (*p* ≥ 0.05).

### 3.3. Indices of Arterial Stiffness

**Figure 3:**
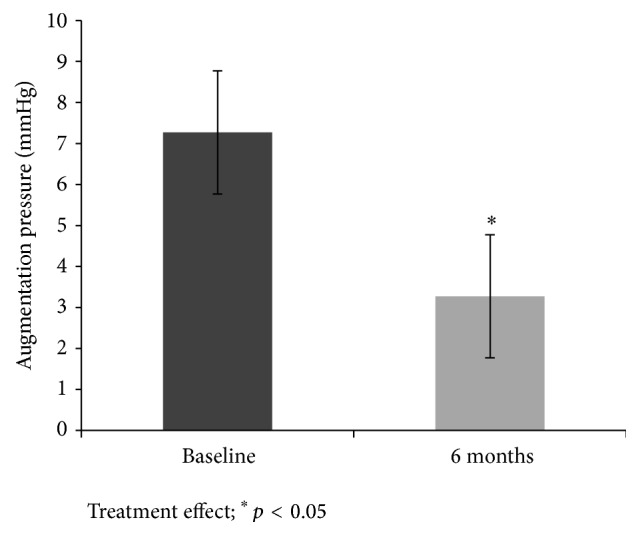
Augmentation pressure (±SD) before and after 6 months of high-dose vitamin D supplementation.

Baseline 25-hydroxyvitamin D was negatively associated with AP, aortic SP, and aortic PP and tended to be associated with PWV (Table 2) (*p* < 0.05). Among the high-dose group, AIx decreased by 12.3 ± 5.3% (*p* < 0.05), whereas there were no similar improvements in AIx among individuals in the low-dose group (*p* ≥ 0.05). However, when AIx was heart rate adjusted (75 bpm; AIx-75), there was no significant effect among individuals in the high-dose and low-dose groups (*p*s > 0.177). Among individuals in the high-dose group, AP decreased by 4.0 ± 1.5 mmHg (Figure 3; *p* < 0.05), whereas there were no similar improvements in AP among individuals in the low-dose group (*p* ≥ 0.05). Decreases in AIx and AP were not correlated with any BP parameter (i.e., clinic or mean 24 hr), even among individuals in the high-dose group (*p* ≥ 0.05). Increases in serum 25-hydroxyvitamin D were not associated with reductions in AIx or AP at 6 months (*p* ≥ 0.05). At 6 months, there were no changes from baseline in any of the indices obtained from PWA (i.e., SEVR, aortic SBP, DBP, and MAP) in individuals within the entire sample, high-dose or groups (*p* ≥ 0.05).

## 4. Discussion

The purpose of the present study was to determine the influence of high-dose and low-dose vitamin D supplementation on markers of arterial stiffness and mean 24 hr BP. Among the total sample, there were no significant differences in mean 24 hr BP over 6 months regardless of vitamin D group. However, high-dose vitamin D (4,000 IU/d) significantly lowered AIx and AP over 6 months of supplementation, with no similar improvements observed in the low-dose group. These results suggest that the pleiotropic, beneficial effect of vitamin D on markers of arterial stiffness may be dose-dependent and that previous inconsistencies in the literature regarding vitamin D and CVD outcomes may be attributable in part to a differential treatment effect of vitamin D dosage. Most notably, these data refute the hypothesis that arterial stiffness moderates BP in individuals with hypertension and vitamin D deficiency as reductions in arterial stiffness were observed* independently* of any significant reductions in BP.

Observational studies have previously shown a negative correlation between vitamin D deficiency and indices of arterial stiffness (i.e., AIx, SEVR, and PWV) [25, 26]. However, few interventional studies have been designed to concurrently investigate the influence of vitamin D supplementation on arterial stiffness and BP. Our findings are in agreement with Al Mheid et al. who observed similar improvements in arterial stiffness and endothelial function after normalization of vitamin D (≥30 ng/dL) in 42 vitamin D deficient (<30 ng/dL) healthy adults, with corresponding pressure changes (mean arterial pressure, MAP: −4.6 ± 2.3 mmHg) [27]. McGreevy et al. also observed significant reductions in median PWV and AIx 8 weeks after a single intramuscular injection of 100,000 IU vitamin D in older adults with vitamin D deficiency (<20 ng/mL), with a corresponding* increase* in SBP [28]. Conversely, Ryu et al. investigated the influence of 2,000 IU/d versus placebo in 45 patients with type II diabetes mellitus and vitamin D deficiency (<20 ng/mL) and found no influence of vitamin D supplementation on arterial stiffness or BP over 24 weeks [29]. Inconsistencies in the existing interventional literature designed to investigate vitamin D supplementation on arterial stiffness are likely due to the inclusion of patients with comorbidities [29] and the use of concomitant medications known to affect arterial stiffness and BP (i.e., antihypertensive therapy) [28, 29] as well as variable doses and duration of vitamin D supplementation [27]. The present study observed beneficial decreases in AIx and AP after 6 months of high-dose vitamin D supplementation. However, these decreases occurred without paralleled reductions in BP, suggesting that hypertension in the presence of vitamin D deficiency is not likely not moderated by arterial stiffening.

The mechanisms by which vitamin D supplementation may reduce certain indices of arterial stiffness likely involve the Renin-Angiotensin-Aldosterone System (RAAS) [30, 31]. Activation of RAS pathways and subsequent increases in vasoconstrictor angiotensin II (Ang II) increase arterial stiffness and vascular tone [26, 32, 33]. Vitamin D receptor knock-out mice experience a marked increase in renin expression, plasma Ang II production, and hypertension [33], while, in adequate levels, 25-hydroxyvitamin D inhibits macrophage stimulation [34] and suppresses endothelin-induced vascular smooth muscle cell proliferation [35], both of which modulate endothelial cell function and arterial stiffness.

In the present study, high-dose, but not low-dose, vitamin D supplementation lowered select indices of arterial stiffness but without paralleled reductions in BP over the course of 6 months. These results indicate that arterial stiffening does not appear to* directly* moderate or influence the relationship between hypertension and vitamin D deficiency. This is perhaps attributable to the fact that hypertension is a multifaceted pathology, as significant improvements in arterial stiffness after high-dose vitamin D supplementation did not appear to even partially mitigate elevated BP. Of note, vitamin D supplementation improved some (i.e., AP and AIx) but not all indices of arterial stiffness. It is unclear why certain indices may respond to vitamin D therapy while others would not. McEniery et al. have reported markers such as AIx to more favorably respond to treatment interventions in individuals <50 yr of age, while individuals >50 yr of age experience reductions in PWV [36]. Resultantly, it has been suggested that, in individuals <50 yr of age, such as the present study (mean age: 37.5 ± 10.9 yr), AIx should be a more relevant marker of arterial stiffness, thus making our findings more clinically intriguing [36]. Nevertheless, it appears as if high levels of vitamin D supplementation (i.e., 6x higher than the Recommended Dietary Allowance) may modulate certain pathways involved in systemic arterial stiffening and warrant further investigation [24].

There are several limitations to the present substudy. First, the present study consists of a post hoc analysis of the larger DAYLIGHT trial [21] and thus was not originally powered to examine arterial stiffness as a major outcome. Second, we lacked key measurements of biomarkers that could possibly explain our findings (i.e., Ang II and renin); therefore, the proposed mechanisms are purely* speculative*. Furthermore, it is possible that the study definition of vitamin D deficiency (≤25 ng/nL) was too high to discern a noticeable effect of vitamin D supplementation on changes in BP or arterial stiffness. However, the overall sample baseline serum 25-dehydroxyvitamin D average was quite low (15.7 ± 6.3 ng/mL), with 93% of the study sample being <20 ng/mL. Even among individuals with large increases in 25-hydroxyvitamin D during supplementation, there was no discernible dose response trend towards lower BP or arterial stiffness. Lastly, decreases in certain indices of arterial stiffness were not related to increases in serum 25-dehydroxyvitamin D levels; thus it is possible that any favorable reductions in arterial stiffness were not related to normalization of vitamin D. However, the lack of correlation is not strong enough to rule out this potential mechanism as measured serum 25-dehydroxyvitamin D levels may not fully explain any observable actions of vitamin D downstream or on a cellular level.

Despite few limitations, the present study possesses several noteworthy strengths. Prior studies examining supplementation with vitamin D used various doses and types and with less rigorous assessments of CVD parameters. We reduced variability in the arterial stiffness and BP response by examining the effect of two daily doses of vitamin D (400 IU/d versus 4,000 IU/d) for 6 months with very stringent assessments. Arterial stiffness assessments were performed by the same researcher to reduce intertester variability. BP assessments were performed according to AHA guidelines in the clinic setting as well as under conditions of daily living using the gold standard for BP assessment (i.e., 24 hr ABPM), again by the same researcher. Finally, to the best of our knowledge, the present study is the largest, randomized, and controlled trial to investigate the influence of high-dose versus low-dose vitamin D supplementation on arterial stiffness in vitamin D deficient individuals with elevated but* untreated* BP. Thus, our findings can be confidently generalizable as our population is representative of similar cohorts who may be prescribed vitamin D therapy but who may not necessarily qualify for antihypertensive therapy according to most recent guidelines [37].

Our findings are supportive of a potential cardiovascular health benefit of high-dose vitamin D supplementation on arterial stiffness. However, reductions in arterial stiffness did not result in positive, corresponding reductions of BP suggesting that changes in arterial stiffness with vitamin D supplementation do not appear to moderate or influence BP in this cohort. Further investigation is needed with a randomized controlled trial intentionally designed to determine the influence of vitamin D supplementation on arterial stiffness among men and women with hypertension to confirm the effects we have observed. The effectiveness of vitamin D supplementation as a monopharmacological or polypharmacological intervention to reduce BP, arterial stiffness, and/or CVD risk is clinically intriguing as it is cost effective and well tolerated and may prove to benefit other conditions.

## Figures and Tables

**Table 1:** Mean baseline characteristics (±SEM) of participants randomized to high-dose versus low-dose vitamin D (*n* = 41).

Variable	Low-dose (*n* = 22)	High-dose (*n* = 19)
Age (years)	34.8 ± 12.8	40.4 ± 7.5^*∗*^
Male (%)	48	52
Body Mass Index (kg/m^2^)	30.5 ± 5.8	32.1 ± 8.7
Serum 25-hydroxyvitamin D (ng/mL)	16.5 ± 6.8	15.1 ± 5.7
Clinic SBP (mmHg)	127.8 ± 5.1	123.7 ± 4.6
Clinic DPB (mmHg)	78.6 ± 1.9	76.9 ± 3.1
Clinic heart rate (bpm)	79.3 ± 2.5	76.7 ± 3.1
24 hr mean ambulatory SBP (mmHg)	125.8 ± 9.9	126.1 ± 9.4
24 hr mean ambulatory DBP (mmHg)	77.5 ± 8.7	78.2 ± 8.0
Daytime ambulatory SBP (mmHg)	128.0 ± 10.4	128.3 ± 8.8
Daytime ambulatory DBP (mmHg)	79.6 ± 8.9	80.5 ± 7.7
Nighttime ambulatory SBP (mmHg)	114.4 ± 10.9	118.5 ± 13.2
Nighttime ambulatory DBP (mmHg)	70.0 ± 10.2	70.0 ± 11.0
Season of enrollment (%)		
Winter	48	47
Spring	23	21
Summer	16	21
Fall	13	11

SBP, systolic blood pressure; DBP, diastolic blood pressure; ^*∗*^ *p* < 0.05; high-dose versus low-dose.

**Table 2:** Relationship between baseline vitamin D and baseline indices of arterial stiffness.

	Partial *r*	*p*
Heart rate (bpm)	.159	0.370
Ejection duration (%)	.288	0.099
Augmentation pressure (mmHg)	−.340	**0.049**
Subendocardial viability ratio (%)	−.013	0.942
Aortic systolic pressure (mmHg)	−.494	**0.003**
Aortic diastolic pressure (mmHg)	−.092	0.605
Aortic mean arterial pressure (mmHg)	−.300	0.084
Aortic pulse pressure (mmHg)	−.575	**0.000**
Pulse wave velocity (m/s)	−.350	0.120
Augmentation index (%)	−.128	0.445
Augmentation index @HR75 (%)	−.084	0.622
